# Carcinoma of the Cervix Uteri1Read at the meeting of the American Association of Obstetricians and Gynecologists, Washington, D. C., September 16, 17, and 18, 1902.

**Published:** 1903-01

**Authors:** L. H. Dunning

**Affiliations:** Indianapolis, Ind.


### SELECTIONS.

### Carcinoma of the Cervix Uteri.[1]

SUMMARY REPORT OF THE AUTHOR'S SIXTY-TWO CASES OPERATED UPON BY HYSTERECTOMY. REMARKS.

By L. H. DUNNING, M. D., Indianapolis, Ind.

(*American Journal of Obstetrics, December, 1902.*)

IN presenting a report of all the cases of cancer of the cervix uteri operated upon up to the date of the present writing—September 1, 1902—the author desires to state that he has excluded from this list all the cases in which a question arose in his mind as to the diagnosis of carcinoma. All but very few of the cases were examined by competent pathologists and pronounced carcinoma, and those not examined presented unmistakable signs of malignancy, such as pain, hemorrhage, and a fetid discharge with the presence of neoplasm which was either fungoid or undergoing ulceration. There were in all 62 cases, and of these 18 are known to be alive. There were 5 deaths resulting from the operation, and one died at the end of ten years and three months.

The list has been divided into two groups, the first group including all cases operated upon previous to September 1, 1898, five years ago. In this group there are 30 cases, of which 5 (16 2-3 per cent.) lived longer than five years. A brief history of these five cases is herewith submitted. By this report it will be seen that one lived ten years and three and a half months. One is alive and well at the end of six years and seven months, one at six years and eleven months, one at six years and four and a half months, and one at five years and nine months.

The second group includes those cases operated upon within the last five years. There are 32 cases in this group. Of these, 14 are still living. One is alive four years and four months after operation, one four years and three and a half months, one four years and one and a half months, two three years and ten months, one one year and ten months, one one year and seven and a half months, one one year and six months, one ten months, one nine months, one eight and a half months, one six and a half months, one five months, and one four and a half months.

1. Read at the meeting of the American Association of Obstetricians and Gynecologists, Washington, D. C., September 16, 17, and 18, 1902.

All of these who have lived over three years have been examined either by myself or attending physician and found to be free from a recurrence. I cannot speak positively of several patients operated recently, other than to say that they do not present at present any symptoms leading to a fear of recurrence.

In this report all cases are excluded in which we knew portions of the cancerous mass were left behind. In our attempts to do thorough work we sometimes jeopardised other tissues. Once a portion was cut out of the bladder and the opening was successfully sutured. Twice we caused rents in the bladder. One occurred in one of the fatal cases, and in the other case the rent was closed by a subsequent operation before the patient left the hospital. The patient died, however, of a recurrence within six months.

In three cases one ureter was severed. In two of them the corresponding kidney was extirpated. In the first, which occurred in 1893, the prognosis was unfavorable and I knew of no better way of procedure. The patient recovered from both operations, but died three months later of recurrence. In the second case I urged an abdominal section, the dissecting out of the ureter and the grafting of it into the bladder. The patient rejected this method in favor of nephrectomy. The patient is still living and seemingly well at the end of two years and eleven months. In the third case of severed ureter a rapid recurrence took place, the open end of the ureter became blocked by cancerous deposits, a hydronephrosis developed, yet the patient lived many months.

In several patients operated upon there was slight extension of the disease to the vagina. If the vaginal involvement is superficial and not more than one inch below the uterovaginal junction, it is no bar to hysterectomy.

The author has several times extirpated the upper portion of the vagina, both by the lower and upper methods. When the work is done through the vagina an incision may be made through the coats of the vagina below the growth, far enough away to enable one to seize with a forceps healthy vaginal tissue. Now, by traction upon the forceps and finger pressure beneath the vagina, between it and the underlying structure, the vagina and neoplasm may be stripped up to the cervix.

In two or three instances I have first severed the vaginal attachment to the uterus and then stripped off portions of the vagina by forceps, traction, and finger pressure from above downward. The former method, however, is the preferable one. The suprapublic method is certainly the preferable one when the uterus and upper portion of the vagina are to be removed.

In all five of the cases that have survived for five years and longer the disease was in its incipiency. There were two, however, in the second group—viz., the one alive at the end of four years and three and a half months and the one alive at the end of four years and one and a half months—in which the disease was so far advanced that I deemed it questionable, at the time of examination, whether the operation would be of any avail. The first was an adenocarcinoma of the cervix and the operation a vaginal hysterectomy. In attempting to draw the uterus down the cervix was torn off, so that the operation was greatly embarrassed. The case proved to be one of the most satisfactory in the list.

The other case was one of a large cauliflower growth of the cervix attended by much bleeding, fetid discharges, and marked emaciation. This patient is now enjoying good health without recurrence. These are exceptional cases. In the review of my experience I am greatly impressed with the necessity of early operation, if we desire to benefit the greater number of patients.

Vaginal hysterectomy was the method of operation in all but five cases. In the five cases in which supravaginal hysterectomy was done, a technique very similar to the one proposed by Werder was employed. The pelvic glands were removed in two cases only. One case out of the five was fatal, and in this case the operation was most difficult and quite prolonged. The left ureter was torn off near the bladder. [See note.] Fortunately

---

[Note.—In a recent supravaginal hysterectomy I resorted to a simple method which yielded me such admirable results that I shall employ it in the future whenever possible.

The case operated upon was one of carcinoma of the cervix in which the neoplasm extended well over toward the right lateral pelvic wall, yet it seemed just above the vaginal tissue, and the uterus and neoplasm moved freely, so I thought it possible to easily extirpate the uterus and neoplasm.

After opening the abdomen and working my way down to the neoplasm, the latter was found more extensive than we at first thought. It seemed to me the ureter was in danger. The patient was in the Trendelenburg position, and the intestines held above the pelvic brim by gauze packing. The ureter, as it came over the brim of the pelvis, was in plain view. The peritoneum lying over it was incised a distance of one-half the torn end of the ureter was found and transplanted into the bladder without difficulty. The result of this implantation we do not know, as the patient died at the end of two days and we were not permitted an autopsy. Only two of the remaining four patients are alive, one six and a half months and one five months after the operation. It seems to me that the question of methods is still an unsettled one.

Certainly the supravaginal operation with extensive dissection of glands and broad ligaments is attended with a larger rate of primary mortality, and the method has not been employed long enough to determine its relative merits in effecting a cure. Theoretically it appears the most promising.

Leopold's researches show that even at a comparatively early stage of the disease too much reliance cannot be placed on this alleged immunity of dissemination, for of 127 cancerous uteri extirpated per vaginam he found dissemination in the parametrium in 68, or 54 per cent., the disease being limited to the uterus in the other 59 cases.

Mackenrodt has arrived at similar conclusions.[1]

Roger Williams's[2] post-mortem findings showed the largest per cent. of involvement of vaginal tissue by extension, viz., 72 per cent. These findings lead us to hope for a greater number of cures when a few years more have passed and we can obtain tabulated reports of cases operated upon by the Werder method. Every supravaginal hysterectomy for cancer of the cervix should include in the procedure the removal of a portion of the upper part of the vagina, especially anteriorly, since this is the most frequent direction of the extension.

1. Roger Williams. Uterine tumors, p. 213.
2. Ibid., p. 224.

---

inch. The peritoneum was dissected off the ureter, a blunt needle threaded with catgut was carried under the ureter, and a single knot was carried down to near the ureter, but not tightened. By slight upward traction it was found to put the ureter on the stretch, so that it could be felt as a cord all the way from the thread encircling it to its entrance into the bladder.

To my satisfaction I thus demonstrated that the neoplasm in the cervix lay below the ureter, and that with a little care there was no danger of wounding it.

When the uterus was extirpated the catgut thread was cut and removed and the incised peritoneum stitched. It seems to me this simple method could be used with marked advantage in many cases of operation upon neoplasms in the pelvis in which there is danger of wounding the ureter.

Vaginal hysterectomy for cancer of the neck of the uterus has yielded me 16 2-3 per cent. of cures, taking the five years time test as the standard of computation. This is a percentage corresponding to that obtained at John Hopkins, as recorded by Cullen in his work, Cancer of the Uterus, (pp. 632 and 633). By consulting his table of cases of squamous-celled carcinoma of the cervix uteri, it will be seen that the whole number operated upon five years previous to the time of his report was 14. From this number two should be deducted, as they were incomplete operations. There are thus left 12 cases operated upon, and 2 of these were alive and well at the time of making the report, thus showing a percentage of recovery of 16 2-3 per cent.

The number of cures is not a complete index to beneficial results of operations for cancer of the uterus. The prolongation of life in many cases, and the relief of suffering, hemorrhage, and offensive discharges, must pass for much in favor of surgery. The writer's experience teaches him, however, positively the uselessness, the harmfulness of hysterectomy in cases in which there is a wide dissemination of cancerous deposits and infiltrations. He has long since abandoned all efforts in this direction. He has, however, seen much temporary benefit result from curettage and the thorough application of the thermocautery.

Of late there has been some discussion upon the relative merits of hysterectomy and high amputation of the cervix in incipient cancer. Several years ago the writer, following the teachings of Schröder, employed high amputation instead of hysterectomy. He abandoned this method because of the incompleteness of the operation, and because of the sufferings of the patients afterward in those operated upon before the menopause. It is probable that amputation yields a slightly less primary mortality than hysterectomy, but the benefits in patients surviving the hysterectomy are much greater.

It should be remembered that in a considerable percentage of cases of cancer of the cervix uteri the body of the uterus is also affected by extension through the lymphatics, and in other instances the disease spreads rapidly from below upward. Obviously amputation of the cervix is inappropriate for such cases. The ovaries and tubes are quite often invaded, even in early cases. Here also amputation is inefficient.

The painful menstruation and pelvic congestion due to stenosis following amputation of the cervix are, as a rule, very distressing and call for frequent intervention of the surgeon. Pregnancy and labor in such cases are distressing and too frequently dangerous.

We operate in cases of cancer to bring relief as well as to accomplish cures. To effect this double purpose hysterectomy is at present the best known means.

REPORT OF FIVE CASES OF CARCINOMA OF THE CERVIX UTERI ALIVE FIVE YEARS AFTER HYSTERECTOMY.

Case I.—Mrs. A., referred by Dr. Terrill, of Filmore, Ind., in January, 1892. The patient was 63 years old and had reached the menopause ten years previously. For several months she had had profuse uterine hemorrhages. Dr. Terrill had removed small growths from the cervix uteri several times, but new ones had quickly sprung from the site of the former ones. At the time of my examination there was a small cauliflower growth springing from the cervix and protruding into the vagina. A portion of it was examined by Dr. Ferguson and pronounced carcinoma. Vaginal hysterectomy with extirpation of the appendages was done January 4, 1892. The patient recovered. She enjoyed good health until May, 1899, when she had a slight stroke of paralysis. She made a partial recovery from this attack and died March 20, 1902, of hydrothorax. No post-mortem was made. There were never any evidences of a recurrence of the carcinoma in the pelvic tissues.

Case II.—Mrs. B., married and aged 30 years, was referred by Dr. J. R. Mauk, Cambridge City. She was emaciated as a result of uterine pain and hemorrhage. A neoplasm was found involving the cervix uteri. Dr. C. E. Ferguson examined a portion of the neoplasm and pronounced it carcinoma. A total extirpation of the uterus and appendages was effected January 30, 1896. The patient recovered from the operation and gradually regained her health. She enjoys reasonably good health and has for a number of years been employed as a stenographer. She has not been examined by me recently, but she makes no complaint indicating a recurrence.

Case III.—Mrs. C., referred by Dr. McNutt, of Connersville, Ind. She was 45 years of age. Examined by me December 2, 1896. She gave the history of frequent and profuse hemorrhages and now has a thin, fetid discharge. Upon examination a small papillomatous growth was found upon the posterior lip of the cervix. A piece was snipped out and examined microscopically and pronounced carcinoma. Vaginal hysterectomy was done December 3, 1896. The uterine appendages were also removed. She made an excellent recovery and today is well. I have examined her recently and find no evidences of recurrence.

Case IV.—Mrs. D., referred by Dr. H. Jameson. The patient was 48 years of age, mother of four children, youngest 17 years. A neoplasm involving one-half of the circumference of the cervix uteri was found upon examination. Microscopic examination showed it to be a squamous-celled carcinoma. Hysterectomy was done April 16, 1896. Patient made an excellent recovery. She reported to me by letter August 16, 1902, as being well and as having no signs of recurrence.

Case V.—Mrs. E., about 40 years of age. Referred by Dr. E. F. Hodges. Patient married and mother of two children. Microscopical examination of cervical neoplasm was made by Dr. Hodges, who pronounced it carcinoma. A vaginal hysterectomy was done October, 1895. Patient was in ill health for some months following the operation. She has been in comparatively good health now several years. The writer examined the patient in July of this year and found a clean vagina with no signs of recurrence.

